# Mycobacterium avium Complex-Related Pulmonary Parenchyma Destruction: A Case Report

**DOI:** 10.7759/cureus.82097

**Published:** 2025-04-11

**Authors:** Aishwarya Viswanath, Arjun Manimaran, Jack Brown, Jhiamluka Solano

**Affiliations:** 1 Internal Medicine, Scunthorpe General Hospital, Scunthorpe, GBR; 2 Internal Medicine, Northern Lincolnshire and Goole NHS Trust, Scunthorpe, GBR; 3 Critical Care Medicine, Northern Lincolnshire and Goole NHS Trust, Scunthorpe, GBR; 4 Resident Doctor Committee, Royal College of Physicians, London, GBR; 5 Education Committee, Academy of Medical Educators, Cardiff, GBR; 6 Cardiology, Scunthorpe General Hospital, Scunthorpe, GBR

**Keywords:** host-directed therapies, mortality factors, mycobacterium avium, non-tuberculous mycobacterial infection, pulmonary disease

## Abstract

Non-tuberculous mycobacteria, particularly Mycobacterium avium complex (MAC), have become significant causes of pulmonary infections, especially in immunocompromised individuals. MAC lung disease presents a diagnostic challenge due to its clinical overlap with other pulmonary conditions, including Mycobacterium tuberculosis infections. The need for microbiological confirmation and radiological evaluation complicates early diagnosis. New molecular diagnostic methods, such as PCR, have improved detection but are not universally accessible. Treatment for MAC lung disease typically involves a multidrug regimen including macrolides, rifamycins, and ethambutol. However, the disease often proves resistant to standard therapies, and treatment failure is common due to drug resistance, delayed diagnosis, and poor adherence. Recent research has highlighted the need for personalized treatment strategies and alternative therapies, such as intravenous antibiotics and host-directed treatments, to improve outcomes. The prognosis for MAC lung disease remains poor, especially in patients with preexisting lung conditions like bronchiectasis or rheumatoid arthritis, who are at increased risk of disseminated infection. This case report and review underscore the importance of early recognition and intervention, highlighting the role of a multidisciplinary approach in managing complex infections. Additionally, it emphasizes the need for continued research to identify more effective treatment options and improve patient outcomes in this growing patient population.

## Introduction

Non-tuberculous mycobacteria (NTM) infections have posed significant logistical and financial challenges to healthcare systems over the past four decades, particularly since the onset of the AIDS (acquired immunodeficiency syndrome) epidemic in the 1980s [1-4]. Clinically, NTM infections are often misdiagnosed as Mycobacterium tuberculosis due to their overlapping presentations, complicating timely and accurate diagnosis [5].

Among NTM pathogens, the Mycobacterium avium complex (MAC) is the most common, accounting for 90% of isolates in NTM-associated lung disease. The clinical course of MAC infections varies widely, ranging from indolent, stable disease to progressive disease requiring treatment, with a five-year all-cause mortality rate of 27% [6,7]. Pulmonary parenchymal destruction is an uncommon manifestation of disseminated MAC infection, with only a few documented cases. Here, we present the case of MAC-related pulmonary lysis, highlighting its clinical significance and implications for disease management.

## Case presentation

In 2023, a 70-year-old male patient, a non-smoker, with a background of bronchiectasis and rheumatoid arthritis, presented to the emergency department due to persistent shortness of breath and cough productive of green phlegm. The patient was known in the community for recurrent chest infections and was being investigated for bilateral cavitating lung lesions and was now under the care of the respiratory team. A bronchoalveolar lavage done a month prior isolated MAC. Regular medications consisted of methotrexate 10mg weekly, folic acid 5mg daily, omeprazole 20mg daily, ferrous sulfate 200mg three times daily, naproxen 500mg daily, and a rescue salbutamol inhaler to use as required.

**Figure 1:**
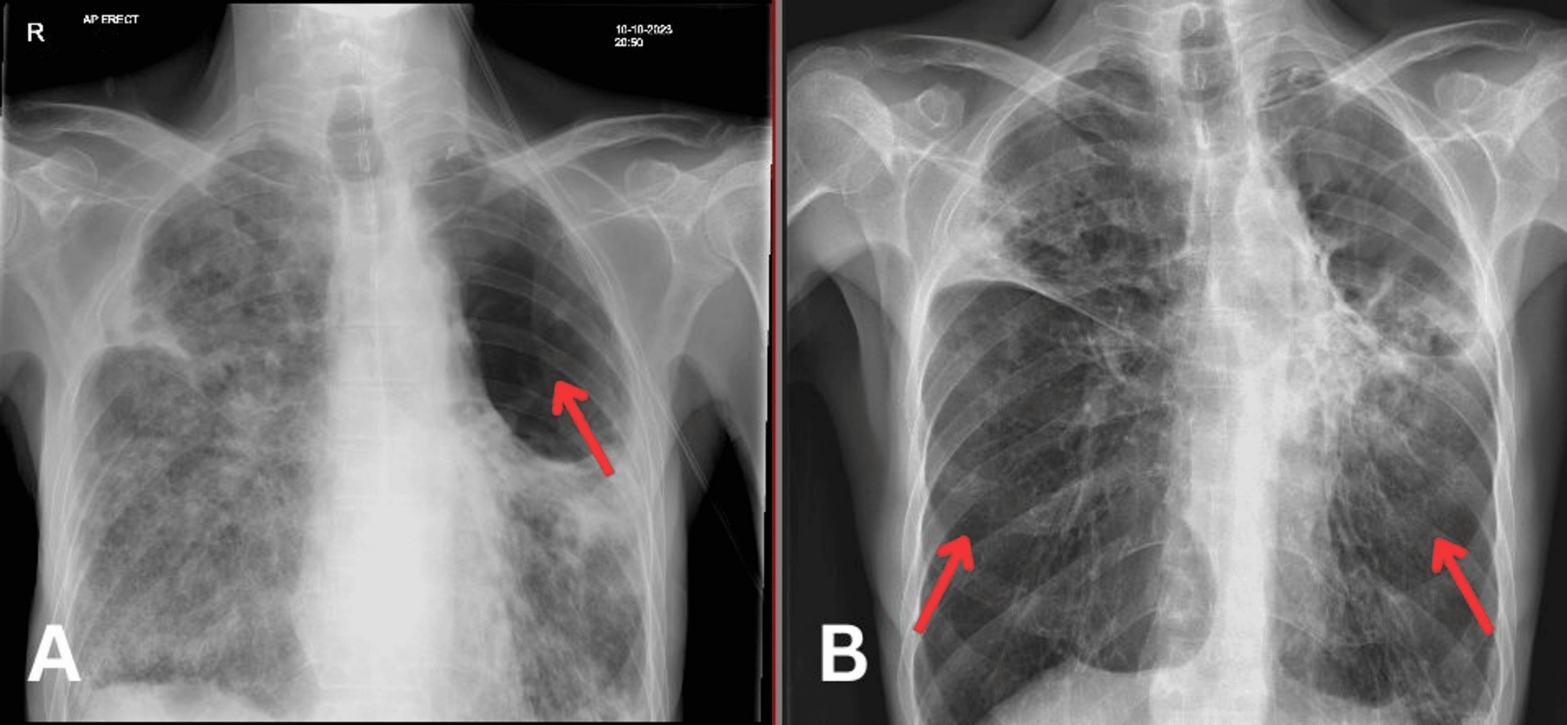
Chest X-ray during the first admission (A) and second admission (B).

On examination, he had a respiratory rate of 32, an oxygen saturation of 96%, a heart rate of 89 bpm, a temperature of 35.5°C, and a blood pressure of 101/62 mmHg. Initial investigations revealed moderate hyponatremia, raised inflammatory markers, and anemia. Chest X-ray showed a large, known cavitation lung lesion in the right upper lobe, a cavity in the left upper lobe, pleural thickening in the left costophrenic angle, and hyperinflated lung fields (Figure 1A). On admission, the patient was diagnosed with atypical pneumonia (with known non-tuberculous Mycobacterium infection) and syndrome of inappropriate ADH secretion (SIADH). He was started on rifampicin 450mg OD, azithromycin 250mg OD, ethambutol 70mg OD, and sodium chloride supplementation. The patient's methotrexate 10mg weekly was stopped indefinitely. He improved clinically and was deemed medically fit for discharge. 

**Figure 2:**
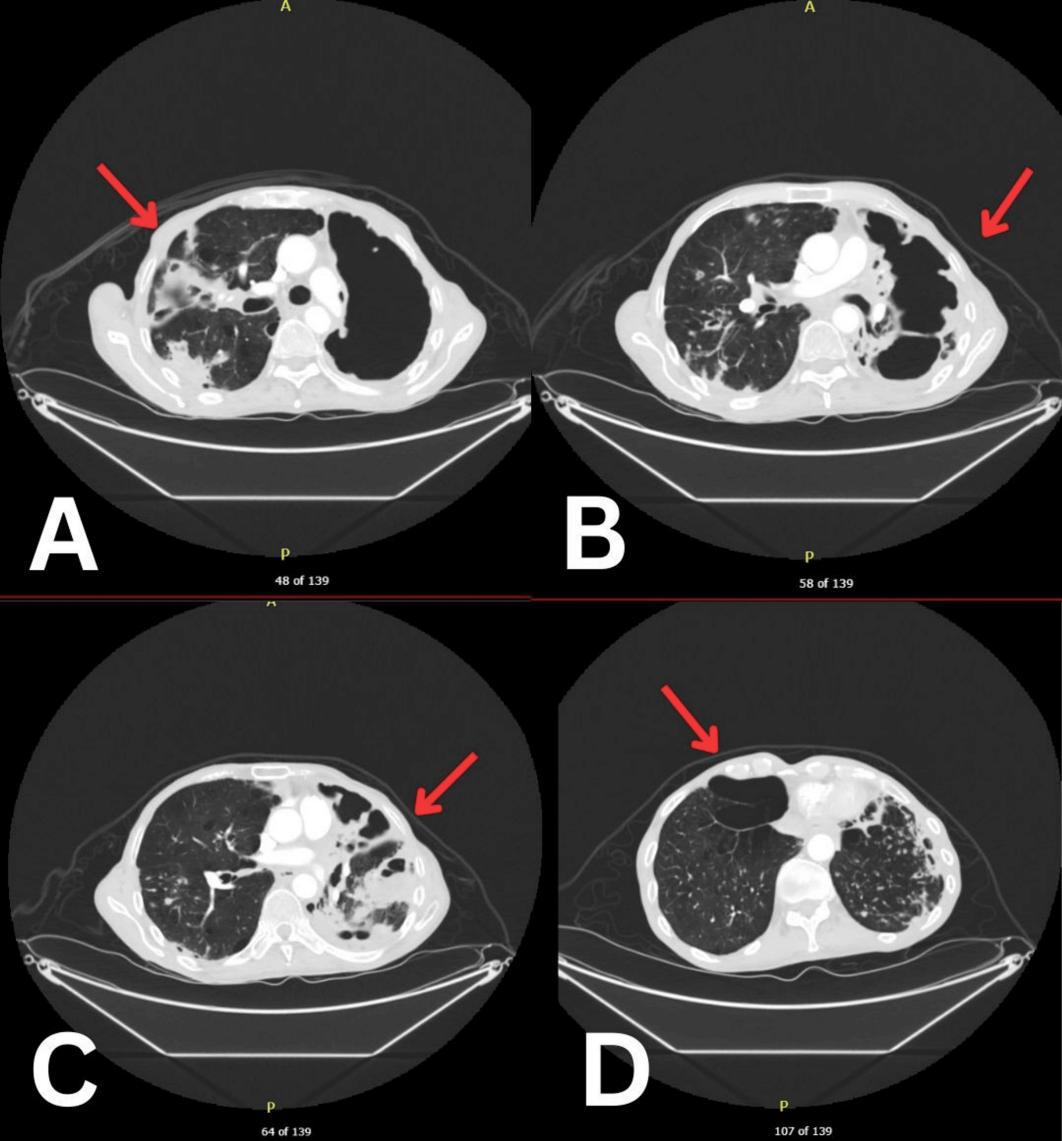
CT-Thorax with views at different levels from cephalic (A) to caudal (D) with left and right-sided cavitating disease (arrows).

Two weeks later, the patient was brought into the resuscitation area of the emergency department by ambulance due to persistent shortness of breath in the community. On admission, he was tachypneic (respiratory rate of 34), and hypothermic at 34.5°C; however, he was able to maintain saturations independently on room air. His initial investigations are shown in Table 1. Chest X-ray (Figure 1B) and CT-thorax (Figure 2) showed worsening right and left-sided cavitating disease. Further investigations for TB, atypical pneumonia screen, and aspergillosis were negative. He tested positive for Mycobacterium avium on a sputum sample. Blood cultures were negative. During admission, the patient experienced several hypoxic episodes registered on pulse oximetry and arterial blood gases. Additionally, the patient showed signs of being malnourished, dropping BMI from 14.07 kg/m^2^ to 13.98 kg/m^2^ which prompted a dietitian review and follow-up. Further follow-up investigations showed dropping albumin levels down to 14 g/L. Unfortunately, despite continuing his rigorous course of antibiotics following local guidance and respiratory support with high-flow oxygen, the patient passed away. The cause of death was established as cavitating pneumonia due to non-TB Mycobacterium infection.

**Table 1:** Admission blood investigations. ALP: Alkaline phosphatase; ALT: alanine transaminase; WCC: white cell count; PLT: platelet count

Investigation	Patient’s results	Normal Value
Sodium	129 mmol/L	133-146 mmol/L
eGFR	>90 mL/min	90-200 mL/min
ALP	274 U/L	30-130 U/L
ALT	7 U/L	0-41 U/L
GGT	210 U/L	10-71 U/L
CRP	176 mg/L	0-5 mg/L
Hb	98 g/L	132-170 g/L
WCC	14.9 10*9/L	4.3-11.2 10*9/L
PLT	661 10*9/L	150-400 10*9/L
Ferritin	598 ug/L	40-405 ug/L

Leading to the admission, the patient had been previously investigated for recurrent chest infections and persistent cough since 2017. Initial investigations revealed a right apical lesion on a CT-thorax. Additionally, bronchoscopy could not rule out malignancy but further confirmed the underlying diagnosis with a positive bronchoalveolar lavage for Mycobacterium avium. The patient was subsequently placed on three-monthly CT follow-ups. In the same year, a bronchoalveolar lavage showed Staphylococcus aureus growth, which was treated following local microbiology guidance. In 2019, a new L upper lobe cavitating lesion was found after a year of stable appearances. By 2021, the patient had suffered a 10kg weight loss in four months, accompanied by a CT report showing growth in the left lung cavity and a new, unexpected left pneumothorax. The patient was subsequently referred for a left lung biopsy.

The biopsy showed extensive active pulmonary fibrosis with no evidence of granulomata or neoplasia. This was deemed to be likely secondary to his rheumatoid arthritis. The lung MDT concluded that the patient was unfit for wedge resection by surgeons due to an increased risk of bronchopleural fistula. As a result, the patient was managed for his recurrent chest infections with antibiotics in the community until his first admission for chest-related symptoms at the end of 2022, where the patient was found to have positive IgEs for aspergillosis. However, the patient was ultimately not diagnosed with allergic bronchopulmonary aspergillosis and was discharged with prophylactic azithromycin with a good response until the reported admission.

## Discussion

This case report, along with a literature review, highlights the complexity of MAC infections in immunocompromised patients. The clinical features and risk factors discussed provide valuable insights into the pathophysiology, diagnosis, and management of these infections. Furthermore, the patient met the clinical, radiological, and microbiological criteria as per the 2017 British Thoracic Society Guidelines for Non-Tuberculous Mycobacterial Pulmonary Disease [8].

The presentation of MAC infections in immunosuppressed individuals, including patients on methotrexate, reflects the interplay between pharmacologic immunosuppression and the body's reduced capacity to combat opportunistic pathogens. Methotrexate's role in impairing immune responses, particularly the suppression of cell-mediated immunity, significantly contributes to the susceptibility to MAC infections. As noted, methotrexate's inhibitory effects on dihydrofolate reductase reduce the proliferative capacity of immune cells, which could potentially facilitate the establishment of MAC infections due to reduced T cell function and cytokine production [9].

It is important to emphasize the high risk of disseminated MAC infections in those with pre-existing structural lung diseases, such as COPD and bronchiectasis, which compromise the lung's defense mechanisms. The presence of RA and its associated interstitial lung disease, compounded by immunosuppressive therapy, creates a heightened risk for severe pulmonary infections. This patient, with a history of methotrexate therapy, underscores the heightened susceptibility to NTM infections, as corroborated by the study in South Korea [10], which revealed an increased mortality rate in RA patients on methotrexate infected with NTM.

The patient's malnutrition, indicated by the BMI of 13.98 kg/m², is a key determinant in the severity of MAC disease progression. A lower BMI, as documented, is associated with a more aggressive form of MAC infection, particularly fibrocavitary disease, which is challenging to treat due to its high bacterial load and poor response to antibiotics. In some cases, surgical interventions can be considered. Furthermore, the presence of cavitary lesions on CT scans, as observed in this patient, signifies an advanced disease state with a poorer prognosis [7]. The compromised vascularity within these cavities hinders effective drug penetration, which significantly contributes to the persistence of infection and subsequent lung damage [11]. The five-year mortality rate for cavitary MAC disease is indeed concerning, as reported by Winthrop et al. [12].

The management of MAC infections in such cases remains a complex task. The delays in diagnostic test results further complicate early intervention. In immunocompromised patients, a prolonged course of multidrug therapy, including clarithromycin, rifampin, ethambutol, and, where necessary, amikacin or streptomycin, is standard [8]. The regimen should continue until cultures are negative for at least one year, a point of crucial importance in achieving a sustained cure. The adjusted therapy regimen for more severe diseases, particularly fibrocavitary disease, provides a clearer framework for personalized treatment strategies.

## Conclusions

This case report provides an essential reminder of the multifactorial nature of MAC infections in immunocompromised individuals. Factors such as methotrexate use, underlying lung disease, and malnutrition significantly affect disease progression and treatment outcomes. Clinicians must carefully consider these factors when diagnosing and managing MAC infections in such high-risk populations. Early detection, coupled with appropriate and sustained antimicrobial therapy, remains critical for improving patient outcomes. Furthermore, clear and good communication with the patient and relatives is key to ensure understanding of prognosis and complications. 
